# A Prospective and Controlled Clinical Trial on Stromal Vascular Fraction Enriched Fat Grafts in Secondary Breast Reconstruction

**DOI:** 10.1155/2016/2636454

**Published:** 2015-12-28

**Authors:** L. A. L. Tissiani, N. Alonso

**Affiliations:** Department of Plastic Surgery, Medical School, University of São Paulo, 04516-010 São Paulo, SP, Brazil

## Abstract

*Background.* Fat grafting is a tremendous tool in secondary breast reconstruction. Stromal vascular fraction (SVF) enriched fat grafts have been presenting promising results regarding volume maintenance. *Methods.* We developed a method that produces a superior SVF enrichment rate (2 : 1) in the operating theatre. This prospective and controlled trial analyzed quantitatively and qualitatively fat grafts with (stem cells group, SG) and without (control group, CG) SVF enrichment in secondary breast reconstruction, through MRI-based volumetry, immunophenotyping, and cell counting. Also, patient satisfaction, aesthetic outcomes, and complications were analyzed. *Results.* Volumetric persistence in the SG was 78,9% and 51,4% in the CG; however it did not reach statistical significant difference. CD90 was the only marker highly expressed in the SG and showed a positive correlation with volumetric persistence (*r* = 0.651, *p* = 0.03). Fat necrosis occurred in 4 patients in the SG and in none in the CG. Patients in the CG showed a trend to be more satisfied. Considering aesthetics, both groups presented improvements. No locoregional recurrences were observed. *Conclusions.* Results are encouraging despite the fact that SVF enrichment in a higher supplementation rate did not improve, with statistical significance, fat graft volumetric persistence. Enriched fat grafts have proven to be safe in a 3-year follow-up.

## 1. Introduction

One of the first descriptions of fat graft was done in 1893 [1] and only a century later did it regain credibility [2]. Coleman published new concepts and an innovative technique to obtain, process, and transfer adipose tissue, which produced consistent and long-lasting results in a variety of fat grafting applications [3–6]. In such a manner, an American survey showed that Coleman's principles were completely or partially incorporated by approximately 50% of the plastic surgeons interviewed [7]. However, many questions about the best technique to handle adipose tissue to be used still remain unanswered.

After Zuk et al. published that the adipose tissue is a rich source of mesenchymal stem cells, regenerative medicine gained an impulse [8–10]. Based on the differentiating capacity the adipose derived stromal cells (ADSCs) present, Yoshimura et al. developed the Cell Assisted Lipotransfer (CAL), the most high-tech type of fat grafting [11]. This technique transforms poor-ADSCs fat grafts into enriched ones, which, in theory, would improve graft take rate and, consequently, volume retention, by stimulating neoangiogenesis and stromal cells differentiation into new adipocytes [12–14]. Some authors have published randomized clinical trials using CAL with favorable and unfavorable results. However they employed different methods of cell obtainment, isolation, and preparation in different clinical settings [15–17]. Recently, De Francesco et al. emphasized that adipose tissue is an important living scaffold for ADSCs, which provides adequate environment for cells to survive [18]. Further, our group, in an* in vitro* model of admixed heterogeneous cell population, showed a positive correlation between the percentage of ADSCs and the increase in* in vitro* adipocyte differentiation [19].

Spear was the pioneer in the use of lipofilling to correct contour irregularities of reconstructed breasts [20] and, since then, a multitude of articles has been published regarding its versatility, safety, and complication rates in aesthetic and reconstructive breast surgeries [6, 21–28]. Likewise, CAL was used in primary breast augmentation [29] and for correcting the sequelae of conservative breast cancer surgeries [30] and congenital deformities [31], but none of these studies was followed by quantitative evaluation. The purpose of this study was that of developing a prospective and controlled trial so as to qualitatively and quantitatively analyze the efficacy of fat grafts with and without a novel type of stromal vascular fraction enrichment as refinements in secondary breast reconstruction.

## 2. Patients and Methods

### 2.1. Ethic Statement

This prospective and controlled study was approved by the Ethics Committee of Faculdade de Medicina da Universidade de São Paulo, Brazil (code 498/11), and was registered at ClinicalTrials.gov under the number NCT01771913. This study was conducted at Carmino Caricchio Public Hospital in São Paulo, Brazil, between March 2012 and May 2015.

### 2.2. Patients

Patients were selected from the Breast Reconstruction Unit in order of presentation, and we started with those in the stem group (SG) followed by the control group (CG). The CG was matched by age, BMI, and radiotherapy with the SG.

The inclusion criteria in both groups were patients with primary breast reconstruction with contour irregularities and BMI between 20 and 35 kg/m^2^, with sufficient fat in the abdomen. Radiotherapy, despite being a confounder factor, was not regarded as an exclusion criterion; however only patients with grades 1 and 2 in the LENT-SOMA scale [32] were included. A stratified blocked randomization was also done to evenly distribute patients with radiotherapy [33]. Patients with breast cancer active disease sequelae of breast cancer conservative treatment, smokers, and uncontrolled comorbidities were excluded.

So as to calculate the sample size, the STATISTICA software required assumptions based on volumetric persistence. In the stem group (SG), the estimated volumetric persistence was considered at 80%, while, in the control group (CG), it was considered at 40%, with a variance of 20% and an alpha error of 5%. Thus, 9 subjects were determined to be allocated in each group (STATISTICA, version 12, StatSoft, Tulsa, USA).

### 2.3. Suction Assisted Lipectomy, Processing, and Lipofilling Surgery

In the operating theater and standing up, patients had their breasts boundaries demarcated and split into four quadrants. Surgeries were conducted under general anesthesia, and autologous fat from abdomen [19] was harvested with a 3 mm cannula with standard low-pressure machine liposuction (−350 mmHg) [34, 35].

In the CG, fat was centrifuged in conic tubes for 2 minutes at 335 g. The intermediate layer was collected and transferred into 3 cc syringes and then grafted with a 1.4 mm blunt cannula in multiple layers mainly in the subcutaneous tissue in a crisscrossed manner.

In the SG, 600 cc of fat was obtained and centrifuged in 50 cc conic tubes for 2 minutes at 335 g. The intermediate layer collected was digested with 1/2 volume of 0.15% collagenase IA (Sigma-Aldrich, MO, USA) for 30 minutes at 37°C with constant homogenization. The aqueous layer was transferred into 50 cc tubes and collagenase was inactivated with 3 volumes of HBSS (Hank's Balanced Salt Solution, Invitrogen, CA, USA). This solution was centrifuged for 5 minutes at 750 g, and the pellets collected were transferred into a sterile bag containing the remainder volume of 300 cc fat centrifugation. The mixture of fat and stromal vascular fraction (SVF) was incubated for 15 minutes under constant homogenization allowing cell adherence to fat to occur. This process resulted in SVF enriched fat tissue at 2 : 1 enrichment ratio.

Supplemented fat grafting was conducted in the same fashion as that of CG. Samples of fat with and without SVF addition were sent to the laboratory for analysis. Considering the time for tissue processing, it took 1 minute to prepare 2.5 cc of fat in the CG while, in the same time period, 2.0 cc of fat was produced in the SG.

### 2.4. Cell Counting

At the laboratory, immediately after surgical procedure, fat graft samples with and without enrichment were digested in the same manner as that at the operating theater. SVF cells were counted and tested for viability using the trypan blue exclusion method in an automatic cell counter (Countess I, Invitrogen, CA, USA).

### 2.5. Immunophenotyping Characterization

So as to assess SVF cells immunophenotype, flow cytometric analyses were conducted in a Guava EaseCyte plus cytometer (Millipore, MA, USA) running the Guava Express Pro 8.1 software.

Freshly isolated SVF from adipose tissue samples (SG and CG) were filtered in 100 *μ*M Nylon Net Filter (Millipore, MA, USA), so as to remove contaminant debris. The sample cells were incubated for 1 hour at 4°C with anti-human CD29-PECy5, CD31-PE, CD34-PerCP, CD45-FITC, CD73-PE, CD90-PE, and CD105-PE (BD Biosciences, NJ, USA). After incubation, labeled cells were washed with phosphate buffered saline (PBS, Invitrogen, CA, USA) and fixed with 1% p-formaldehyde (Sigma-Aldrich, MO, USA). Analyses were conducted on 5 × 10^3^ labeled cells per sample for each antibody, and nonlabeled cell samples were used as control. Laboratory personnel were blinded to the sample analysis.

### 2.6. Breast Volumetry

**Figure 1:**
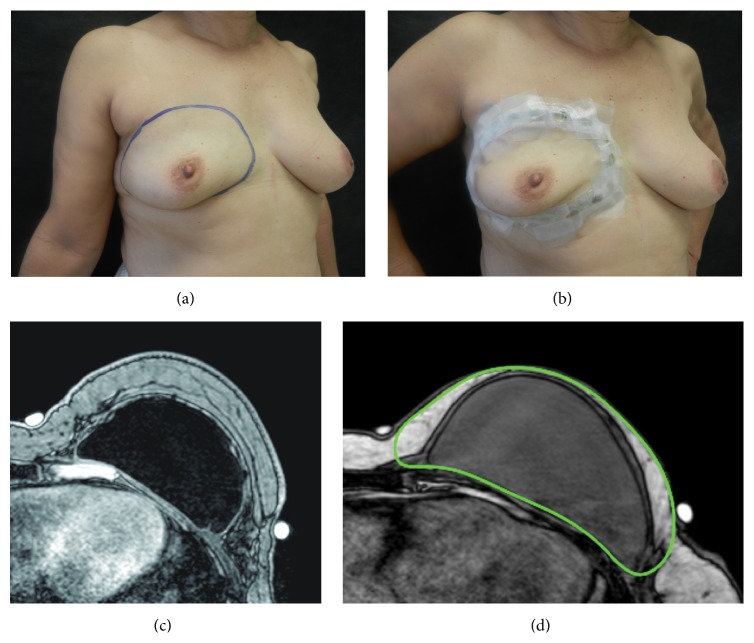
Method for MRI-based breast volume measurement. (a) Breast boundaries demarcated, (b) external markers applied, (c) axial sequence showing the breast medial and lateral limits, and (d) a selected region of interest (ROI) with the OsiriX software.

Patients were MRI scanned without previous injection of gadolinium contrast, and, 6 to 19 months after the lipofilling surgery, they were scanned again with the purpose of determining the breast volume. A 1.5 Tesla scanner (Inthera, GE, Contagem, Brazil) was employed with 3 mm thick slices. We developed a new strategy for determining and computing the boundaries and volume of a reconstructed breast in a more precise way. Just before the MRI exam, the senior investigator marked the boundaries of each breast. With a dermographic marker, a line was drawn throughout medial, lateral, inferior, and superior breast limits. Vitamin E capsules (external markers) were applied on skin over the line, so as to allow more precise regions of interest (ROI) to be determined, and performed on axial sequences by an independent radiologist (Figure 1). OsiriX software, 32 bits, free version (Pixmeo, CA, USA) was utilized to calculate breast volume. Two calculations were done per exam and the average determined was taken as the final breast volume.

### 2.7. Patient Satisfaction Assessment

A patient satisfaction survey was conducted for this study. We included a modified Michigan's questionnaire [36], a visual analogue scale with 5 possibilities (very unsatisfied, unsatisfied, neither unsatisfied nor satisfied, satisfied, and very satisfied), and a score scale ranging from 1 to 10 to assess the final breast aesthetic result. Patients from both groups answered the satisfaction questionnaire at the time of the postoperative MRI scan.

### 2.8. Aesthetic Results Evaluation

Five plastic surgeons who were not involved in the conduction of the study and had different types of breast reconstruction expertise were invited to objectively and independently analyze improvements in breast contour. Panels containing blinded frontal pre- and postoperative photos were prepared for analysis. Surgeons were able to choose 5 different situations: strongly worse, mildly worse, no change, mild improvement, and strong improvement. For each score, a value was attributed as follows: −2, −1, 0, +1, and +2. For analysis purposes, the sum of all five scores, per patient, was taken as the final value.

### 2.9. Clinical Events

Patients of both groups were monitored for the occurrence of adverse events of any type, locoregional cancer recurrences, fat necrosis, oil cysts formation, skin necrosis, and infection.

### 2.10. Statistics

The data gathered was analyzed by means of the R Statistical Software, version 2.15.2 (R Foundation, Vienna, Austria). The data is expressed by mean (range and standard deviation), median (range), and percentages. Comparison between groups was done with Student's *t*-test or Mann-Whitney for age, BMI, breast volumetry, fat graft volume, time of follow-up, basal cell counting, question 6 of the self-assessment questionnaire, and surface markers expression. Wilcoxon was used to compare the number of cells in the pellets before and after enrichment with SVF cells. Fisher exact test was employed to analyze radiotherapy distribution, occurrence of fat necrosis, and questions 2, 3, 4, and 7 of the self-assessment questionnaire.

## 3. Results

Eleven patients were recruited for the SG and nine were recruited for the CG. However, one patient withdrew her informed consent, and the CG finished with 8 participants. Patient demographics are shown in Table 1.

**Figure 2:**
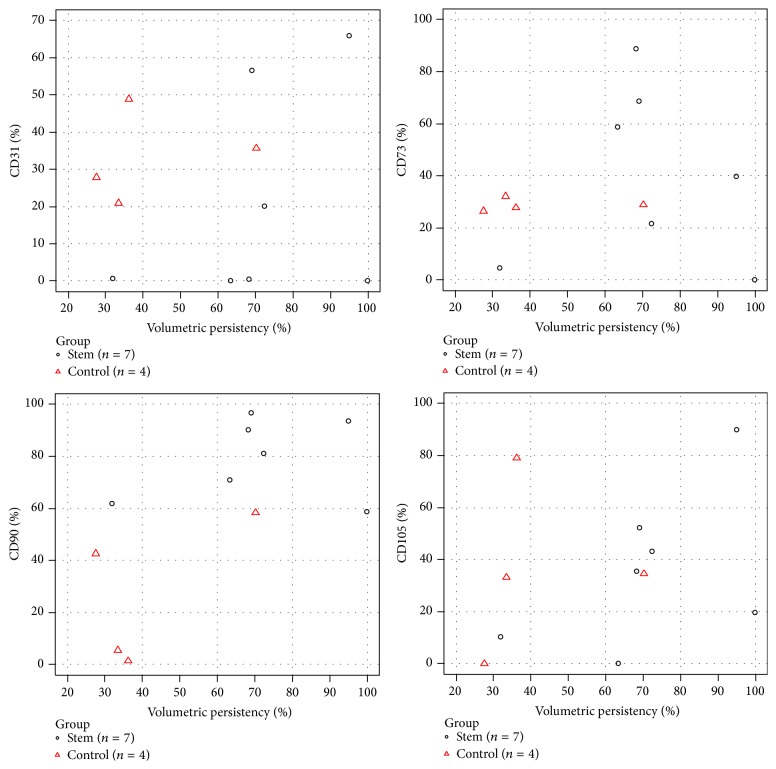
Charts show a positive correlation between surface markers expression and volumetric persistence for CD31, CD73, CD90, and CD105.

The method developed by the authors (2 : 1 enrichment rate) and used to boost the fat grafts in the SG produced an enrichment of 2.6-fold the number of basal cells (*p* = 0.005) (Table 2). Expressions of cell surface markers done in the fresh SVF are shown in Table 3 and a wide variability in their expression was observed among all patients. Taking both groups together, CD45 was the least expressed, while CD29 and CD90 were the most expressed. However, the mesenchymal cell marker CD90, highly expressed in the SG, was the only marker that reached a statistically significant difference among all (*p* = 0.026). There seemed to be a positive correlation between CD31, CD73, CD90, and CD105 expressions and volumetric persistence; however CD90 was the only marker that showed significance (*r* = 0.651 and *p* = 0.03) (Figure 2).

Volumetric persistence in the SG was higher (78.8%, SD = 74.9) than that in the CG (51.4%, SD = 18.4); however, it did not reach a statistically significant difference (*p* = 0.31). Fat necrosis was present in four patients in the SG and in no patients in the CG (*p* = 0.103) (Table 4). Fat necrosis was surgically removed and the pathological findings confirmed this diagnosis for 3 patients. One patient was observed and the ultrasound follow-up showed no need for intervention.

In the long-term follow-up of both groups, no adverse events of any type, no infections, no skin necrosis, and no locoregional recurrences were observed.

The analysis of the satisfaction assessment questionnaire showed that all patients in both groups would choose to undergo breast reconstruction, and they were sufficiently informed about the fat grafting procedure. In both groups, the vast majority of patients were satisfied with the results of fat grafting (*p* = 0.603), would undergo the fat grafting procedure again (*p* > 0.999), and would recommend the fat grafting procedure to a friend (*p* = 0.546). When patients were allowed to freely give a score to their cosmetic result (self-assessment), scores ranged from 5 to 10 in SG and from 8 to 10 in CG (*p* = 0.075). These results show a strong trend in patients of the CG to be more pleased than patients in the SG. When satisfaction was evaluated through a visual analogue scale, patients of both groups were similarly satisfied (*p* = 0.52).

**Figure 3:**
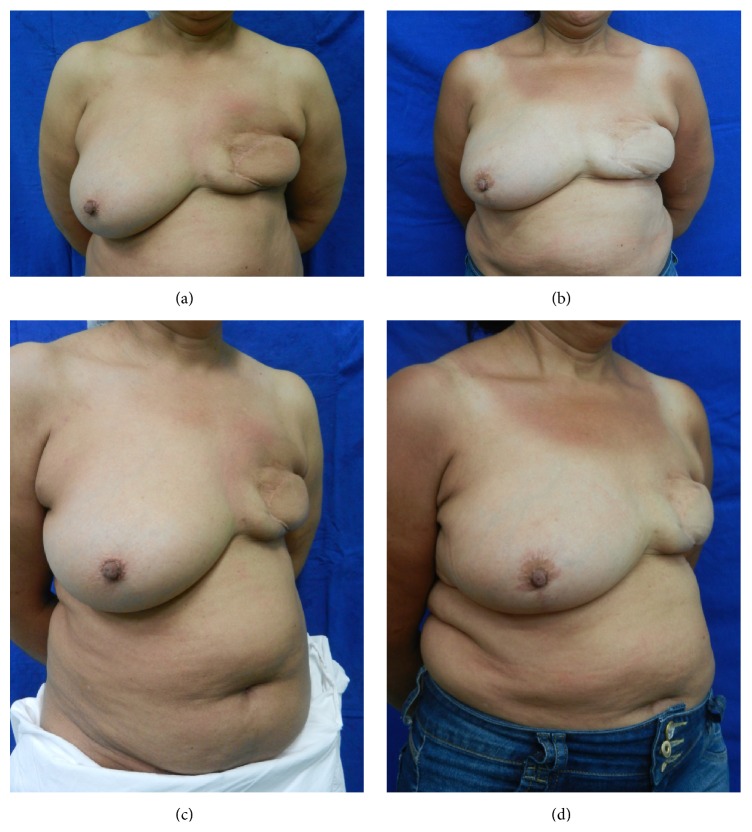
(a, c) Preoperative frontal and oblique views of a CG patient in which 147 cc of fat was injected; (b, d) 12-month postoperative frontal and oblique views with volumetric persistency of 41.6%.

**Figure 4:**
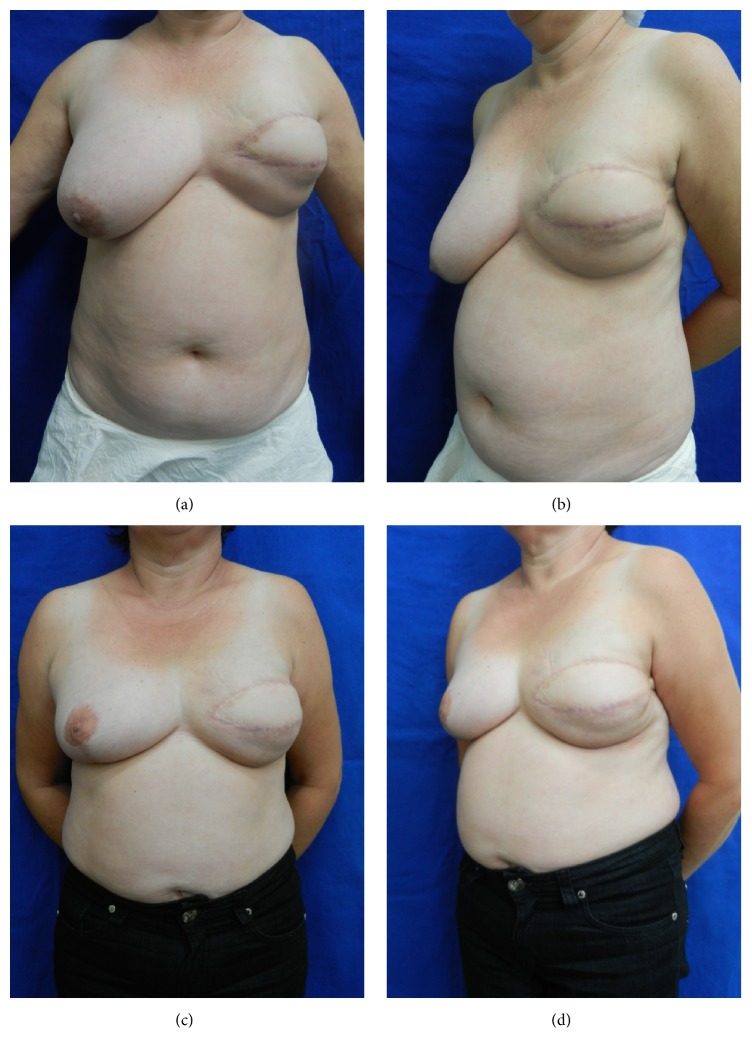
(a, b) Preoperative frontal and oblique views of a SG patient in which 141 cc of enriched fat was injected; (c, d) 12-month postoperative frontal and oblique views with volumetric persistency of 69.1%.

Initially, the 5-peer analysis showed disagreement in the pair-to-pair comparison and in the general comparison, with low values of kappa coefficient. So, changing the 5 subsets into 3 (worsened (−1), nothing changed (0), and improved (+1)), surgeons agreed to a minor degree (kappa = 0.131, confidence interval = 0.020; 0.242). Figures 3 and 4 show patients that were categorized as showing “improvement” by all peers. When computing the new scores, patients in the SG and in the CG received the respective scores (average) of 2.9 and 2.3 (*p* = 0.60) and, therefore, were regarded as presenting similar improvement.

## 4. Discussion

Taking into account age, BMI taken before and after fat grafting, time which elapsed between MRIs, and radiotherapy distribution, the groups are statistically similar. At the very beginning of the study design, in taking into account the sample size and radiotherapy as confounder factor, the stratified block randomization allowed an even distribution [33]. The high incidence of radiotherapy represents the great majority of patients seeking delayed breast reconstruction, and this is corroborated by other publications [37, 38]. The effects of radiotherapy on fat graft retention still are controversial. Rigotti et al. showed the damage to the microcirculation caused by radiotherapy and the benefits fat grafting promoted, including progressive regeneration and neovessel formation [39]. Khouri et al. recently showed that breast reconstruction after radiotherapy needed an average of 4.8 procedures compared to the 2.7 ones for the nonirradiated group [40], and this is in accordance with the work of Losken et al. [24] and, more recently, with the paper published by Longo et al. [41]. In turn, de Blacam et al. [25] showed the same rate of complications when fat grafting was used in secondary breast reconstruction with and without radiotherapy. Choi et al. published the same fat graft volume retention rate in reconstructed breasts with and without radiotherapy [42]. Regarding volumetric persistence and the incidence of complications, the present study showed no difference between the patients who had received radiotherapy and those who had not before fat grafting.

In our study, two patients in the SG behaved as outliers for volumetry. Patient 6 had a TRAM flap reconstruction and put on weight, 6 kg, by the time the postoperative MRI scan was conducted. According to Gutowski et al. [43], fat grafts may show volume change with weight fluctuation and, by taking into account that all patients with TRAM flaps in this series had high volumetric persistence rates and in this patient's case in particular, the weight gain might be responsible for the high volume determined. Patient 1 was irradiated and received the smallest volume of fat grafting. Considering the volumetric loss that all fat grafting may undergo and the internal error of the volumetric calculation tool in the pre- and postoperative MRI scan, the final volume ended up negative.

Inspired by Yoshimura et al.'s previous publications [11, 44], we developed a method that produced an enrichment rate (2 : 1 ratio) that is higher than CAL (1 : 1 ratio), which could be reproduced in the operating theater by other investigators with no difficulties. Based on previous papers and assumptions [13–15, 45, 46], the idea of adding more SVF cells into a fat graft that could render a better biological framework and warrant more volumetric persistence in the long term, considering that there would be more mesenchymal cells to differentiate into new adipocytes and secrete a greater amount of trophic factors, such as proangiogenic and antiapoptotic factors [47, 48], sounded appealing. However, despite all the authors' efforts to produce a substantial enrichment, the volumetric persistence in the SG did not reach a statistically significant difference when compared to that in the CG.

As much as we know, there is no published prospective and controlled study that objectively measured breast volumetry in the field of breast reconstruction using SVF enriched fat grafting. Our results are very optimistic since volumetric persistence as high as this one was only reported by Kølle et al. [15], who achieved persistence of 80.9% in the study group versus 16% in the control group, and Tanikawa et al. [16], who used CAL for the correction of craniofacial anomalies and achieved 88% of volumetric persistence in the study group when compared to 54% in the control group. The former employed a super enrichment rate with cultured-expanded cells injected in the arm, while the latter used manual CAL for correcting soft tissue defects associated with craniofacial microsomia. Concerning cell enrichment ratio, our study relies somewhere between these previous studies, and the answer to explain our results may be the variability in the type of breast reconstruction techniques, which present different amounts of scar tissue in the recipient bed.

Conversely, Choi et al. [42] used 3D-imaging volumetry to analyze volumetric persistence of centrifuged fat grafts, without enrichment, in secondary breast reconstruction. They found an average of 42% volume retention at 140 days after surgery, which is regarded as a short-term follow-up when it comes to fat grafting volume persistence. The RESTORE-2 study [30] and the study conducted by Gentile et al. [31] employed enriched fat grafting by means of the Celution system (Cytori, San Diego, USA) in secondary breast reconstruction, and, despite the good results published, these studies do not possess objective volumetry. Yoshimura et al. [49] used CAL as rescue for breast implant complications and had volume retention between 40 and 80%, and the main criticism to their study is that it does not have a control group.

Likewise, Peltoniemi et al. [17] used enriched fat grafts for primary breast augmentation. Patients of both groups in this controlled study had an average of 50% volumetric persistence, a similar retention rate obtained by other authors, who did not employ stromal cells enrichment but used the BRAVA system [50, 51]. Comparatively to Peltoniemi et al. work, Spear and Pittman [52] showed 39% volume retention in primary breast augmentation with conventional centrifuged fat grafting, and, based on the results published by Khouri and others [21, 23, 51], they drew attention to preoperative breast external expansion as a method of improving some important aspects of the recipient bed, such as neoangiogenesis and a favorable interstitial pressure, before cosmetic breast augmentation with adipose tissue.

The immunophenotyping of the fresh stromal vascular fraction in this study showed a similar surface marker profile compared to that published by Matsumoto et al. [46]. The great majority of studies have published immunophenotyping of the stromal vascular fraction cells after at least one expansion, and our study focused on the analysis of the fresh SVF. Thus, we could observe that the flow cytometric analysis showed a very individualized profile of surface markers expression. In such a way, no patient presented a similar profile. Patients in the SG expressed more CD90 than patients in the CG, and there was a positive correlation between the expression of CD90, a typical mesenchymal marker, and volume persistence. Meanwhile, patients in both groups, who presented high volumetric persistence, demonstrated high CD90 expression.

Modified Alderman's questionnaire showed the importance of breast reconstruction after mastectomy. Moreover, secondary breast reconstruction with fat grafting, with or without stromal cells enrichment, promoted a high level of patient satisfaction. Patients in the CG tended to become more satisfied than the patients in the SG, and the explanation for that is the incidence of fat necrosis that caused distress regarding local recurrence and led to reoperation in 3 patients. Fat necrosis only occurred in the SG and in patients who received radiotherapy. We speculate that even though stromal cells are more resilient to hypoxia and were present in greater number than that in the CG grafts, in some cases, together with mature adipocytes, they were not able to survive the hostile recipient bed, marked by intense fibrosis secondary to radiotherapy and surgical manipulation and damaged microcirculation [39, 45]. Another possible explanation is that the marked fibrosis present in the recipient bed could have misconducted these cells to another path of differentiation contributing to the formation of small nodules of fat necrosis [53]. Similarly, Yoshimura et al. [11] reported two cases of focal fibrosis on thorax and breasts when they injected SVF cells suspended in saline just after injecting fat for cosmetic breast augmentation. They discussed the possible absence of signaling from the adipose tissue, reassuring the importance of employing it as a vital living scaffold [53].

The kappa coefficient showed a weak agreement among raters; however, the evaluation of aesthetic results was positive, meaning that contour irregularities were improved by the fat grafting procedure in patients of both groups. In the complete follow-up, patients have not presented infection, skin necrosis, or any donor site morbidity. In our study, despite not intending to be a long-term follow-up outcome, locoregional recurrences have not emerged in an average follow-up of 16 months in the CG and 36 months in the SG, and this data may contribute to the existing literature about ADSCs enriched fat grafting safety in secondary breast reconstruction. Three patients, 1 from the SG and 2 from the CG, were diagnosed with DCIS at the time of mastectomy, but none of them fulfilled the requirements published by Petit et al. [54] in a way to be considered as being at a higher risk for local recurrences. However, these patients still are under a regular and watchful follow-up. Our findings regarding oncological safety are in agreement with others previously published [43, 55–57].

Limitations to this study include the high incidence of radiotherapy among patients and the absence of randomization, which is justified by the fact that this study was carried out in a single breast reconstruction unit without a large number of patients requiring refinements to be randomized in each group.

## 5. Conclusions

The results of this study are encouraging despite the fact that enrichment of fat grafts with SVF cells at a 2 : 1 proportion did not present a better volumetric persistence rate in the secondary breast reconstruction scenario. A real time higher supplementation rate of fat grafts with SVF cells, without expansion, can be done in the operating theater if appropriate material and personnel are available. Considering an average follow-up of 3 years, the enrichment of fat grafts with SVF cells has proved to not promote locoregional recurrences. The incidence of fat necrosis raises concerns over enriched fat grafts at a 2 : 1 proportion, and they may not be suitable for patients who have previously received radiotherapy. The adequate enrichment rate to ensure a higher volumetric persistence is to be determined by future studies.

## Figures and Tables

**Table 1:** Patient demographics.

	Age	BMI before	BMI after	Types of tumor	Type of reconstruction	Time from mastectomy to fat graft	Graft follow-up	Time between MRIs	RTX
Stem group									
1	55	24,2	24,0	LCI	LD + IMPL	3 y 8 m	4 y 1 m	12 m	Yes
2	47	25,4	25,8	Mucinous	LD + IMPL	3 y 9 m	3 y 9 m	11 m	Yes
3	49	28,0	27,6	DCI	EXP + IMPL	3 y 2 m	3 y 2 m	15 m	No
4	48	27,2	27,6	DCI	LD + IMPL	3 y 7 m	3 y 2 m	15 m	Yes
5	51	28,7	28,7	DCI	EXP + IMPL	2 y 6 m	3 y 1 m	13 m	No
6	41	27,5	29,1	DCIS	TRAM	4 y 10 m	3 y 1 m	16 m	Yes
7	54	24,0	25,1	DCI	LD + IMPL	3 y	2 y 10 m	13 m	Yes
8	44	23,5	23,3	DCI	LD + IMPL	4 y 1 m	2 y 9 m	16 m	Yes
9	56	23,9	23,9	DCI	TRAM	4 y	2 y 9 m	19 m	Yes
10	58	25,6	25,6	Nontumor	No reconstruction	20 y	2 y 6 m	13 m	No
11	43	30,9	29,7	LCI	Seq explantation	4 y 1 m	1 y 8 m	17 m	Yes

Average						**5 y 2 m**	**36 m**	**14,5 m**	**72,7%**

Control group									
1	51	29,2	29,2	DCI	EXP + IMPL	3 y 2 m	2 y 6 m	16 m	No
2	40	20,8	20,4	Medullar	EXP + IMPL	3 y 1 m	1 y 11 m	19 m	No
3	56	32,4	32,6	DCI	Seq explantation	7 y 3 m	1 y 9 m	19 m	Yes
4	69	25,9	28,1	DCIS	LD + IMPL	16 y 6 m	1 y 3 m	13 m	Yes
5	38	24,1	26,1	DCI	LD + IMPL	2 y 5 m	1 y 1 m	11 m	Yes
6	36	24,1	23,2	DCI	LD + IMPL	8 y 3 m	1 y	11 m	Yes
7	59	25,6	25,2	LCI	EXP + IMPL	5 y 8 m	8 m	7 m	No
8	49	24,9	24,3	DCIS	Seq explantation	2 y 8 m	8 m	7 m	Yes

Average						**6 y 1 m**	**16 m**	**12,9 m**	**62,5%**

*p* values	0.977	0.765	0.861	nm	nm	nm	nm	0.414	>0.999

BMI, body mass index; LD, latissimus dorsi; Impl, implant; Seq, sequelae; DCI, ductal carcinoma invasive; DCIS, ductal carcinoma in situ; LCI, lobular carcinoma invasive; Exp, expander; m, months; RTX, radiotherapy; nm, not measured; y, years.

**Table 2:** Basal and after-enrichment cell counting in the SG.

	Basal cell counting	Cell counting after enrichment^*∗*^	Cellularity shift
*n*	10	11	10
Average	524.760,0	1.108.818,2	679.940,0
Median	175.000,0	400.000,0	390.000,0
Minimum–maximum	21.600–2.500.000	42.000–6.400.000	20.400–3.900.000
SD	791.542,2	1.829.290,4	1.147.997,9

^*∗*^Wilcoxon *p* = 0.005.

**Table 3:** Surface markers expression in both groups (SG and CG).

	CD29 (%)	CD31 (%)	CD34 (%)	CD45 (%)	CD73 (%)	CD90 (%)	CD105 (%)
Stem group							
*n*	7	7	5	7	7	7	7
Average	70,09	20,56	43,92	8,59	40,36	79,01	35,81
Median	80,54	0,74	18,64	1,6	39,78	81	35,52
Minimum	21,78	0	11	0	0	58,86	0
Maximum	94,9	65,92	92,3	32,5	88,88	96,66	89,78
SD	28,34	28,86	41,39	12,38	33,55	15,37	30,11

Control group							
*n*	4	4	4	4	4	4	4
Average	75,37	33,34	38,94	15,42	28,84	26,99	36,71
Median	73,88	31,78	45,23	0,3	28,3	24,09	33,88
Minimum	56,48	20,9	0	0	26,54	1,38	0
Maximum	97,24	48,9	65,3	61,08	32,2	58,38	79,08
SD	19,69	12,01	28,2	30,44	2,44	27,95	32,45

Total							
*n*	11	11	9	11	11	11	11
Average	72,01	25,2	41,71	11,07	36,17	60,09	36,14
Median	80,54	20,9	38,32	0,62	28,86	61,82	34,6
Minimum	21,78	0	0	0	0	1,38	0
Maximum	97,24	65,92	92,3	61,08	88,88	96,66	89,78
SD	24,6	24,18	34,09	19,54	26,66	32,64	29,33

*p*	0,751^a^	0,315^b^	0,905^b^	0,527^b^	0,400^a^	0,026^a^	0,964^a^

^a^ *t* -Student for independent samples, ^b^Mann-Whitney.

**Table 4:** Volumetry and fat graft complications.

	Breast volume	Graft	Breast volume	Volumetric^*∗*^	Fat
	Preoperative (cc)	Volume (cc)	Postoperative (cc)	Persistence	Necrosis^*∗∗*^
Stem group					
1	630.45	45	618.15	−27.33%	Yes
2	527.11	92	589.83	68.17%	Yes
3	623.22	177	735.37	63.40%	No
4	885.96	137	1.005.43	87.20%	Yes
5	686.93	180	857.93	95.00%	No
6	1.048.12	147	1,454.60	276.51%	No
7	702.85	141	800.25	69.07%	No
8	547.30	117	584.71	31.97%	Yes
9	732.20	111	842.97	99.79%	No
10	254.14	171	377.69	72.25%	No
11	512.32	159	562.22	31.38%	No

Average		**134.3**		78.8%^*∗*^	36,4%^*∗∗*^

Control group					
1	674.58	96	732.6	60.50%	No
2	470.92	75	525.57	72.86%	No
3	383.83	147	447.55	41.63%	No
4	785.21	111	861.3	68.60%	No
5	603	108	678.85	70.23%	No
6	552.97	115	584.7	27.65%	No
7	778.7	138	824.9	33.50%	No
8	253.25	102	290.2	36.20%	No

Average		**111.5**		51.4%^*∗*^	0%^*∗∗*^

^*∗*^Mann-Whitney *p* = 0.301; ^*∗∗*^Fisher exact *p* = 0.103.
